# Supplementary material for: Texas 2-step: a new model for YcgR::c-di-GMP action at the flagellar motor
Source: J Bacteriol. 2025 Nov 26;207(12):e00353-25. doi: 10.1128/jb.00353-25 (PMC12713396; doi:10.1128/jb.00353-25)
Supplement: Supplemental figures — Figures S1 and S2. [file jb.00353-25-s0001.docx]

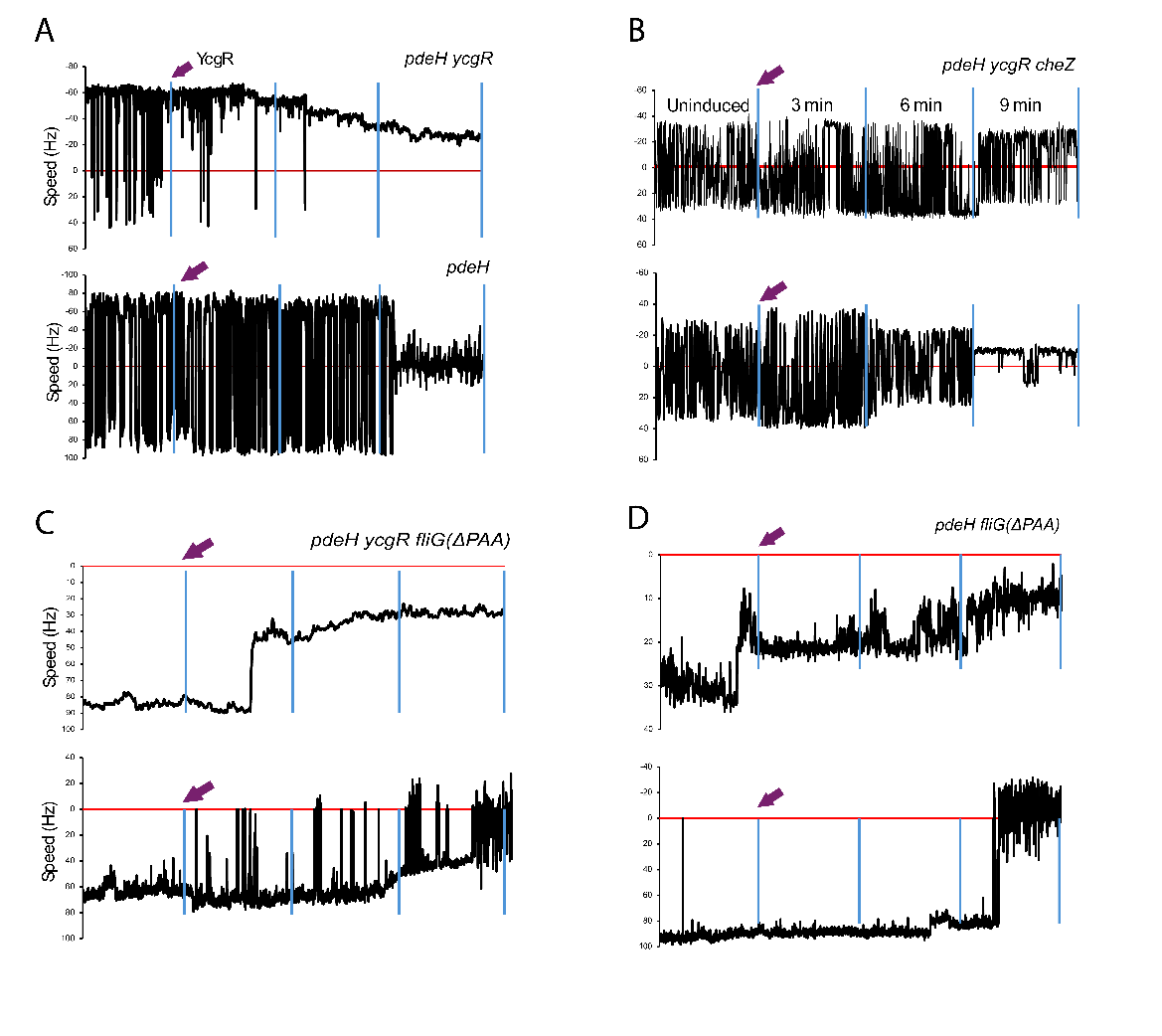
**Supplementary Figures and Movie Legends**

**Fig S1.** Behavior of motors in varied backgrounds and bead sizes. **A.** Two representative motors from WT** MG1655 strain (NBN48). All other descriptions as in Fig 2A. **B**. Behavior of two *cheZ* motors (NBN47) when the bead size was 0.99 μm. **C,D.** Representative *fliG* (ΔPAA) motors in WT* (NBN121) and WT** (NBN89) backgrounds. Bead size was 0.75 μm. Other descriptions as in Fig. 3A.


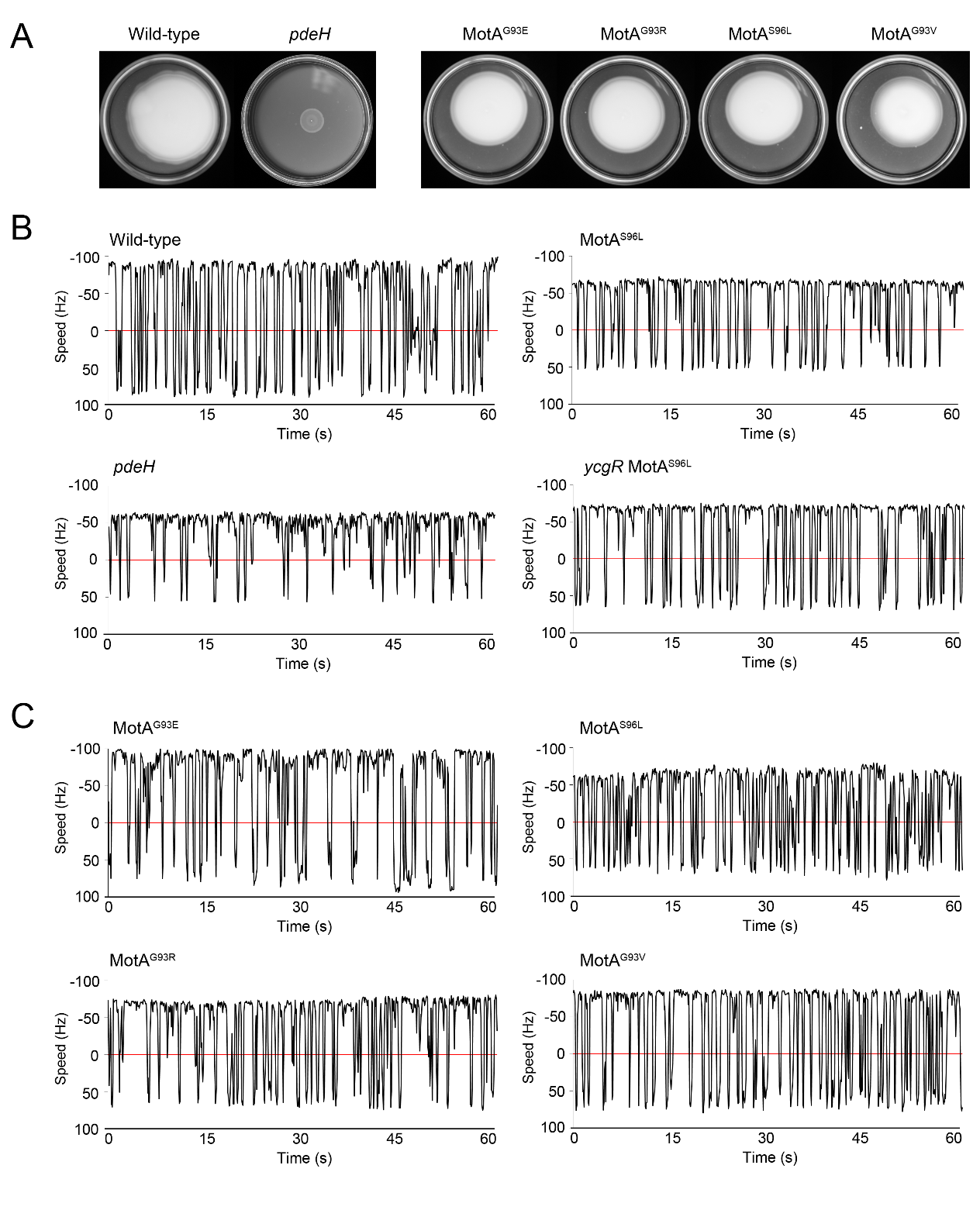


**Fig. S2**. Swimming and motor behavior of MotA suppressors. **A.** Motility assays were conducted in soft agar (see Methods). Left panel, motility is inhibited in a *pdeH* mutant (WT, MG1655; *pdeH*, AB607). Right panel, four MotA suppressors that overcame the motility defect of the *pdeH* mutant, described by Boehm et al. (see Table S1 for strain numbers). **B.** Bead assays in WT and indicated MotAS96L suppressor (AB1577) in which *ycgR* was deleted (JP1501). **C.** Bead assays in four MotA suppressor strains shown in A. The WT strain is MG1655 for all motors, monitored with 0.75 μm polystyrene beads. Representative traces of 15 motors each are shown.

**Movie 1**. YcgR interaction with CCW stator–C-ring complex, with the two YcgR domains straddling MotA and FliG_C_. The animation was created using ChimeraX 1.9 and assembled in Keynote. Structural models used include YcgR (PDB: 5Y6F), CCW C-ring (PDB: 8UMD), and MotA (PDB: 6YKM).

**Movie 2.** YcgR interaction with CCW stator–C-ring complex, with YcgR binding FliG_N,_ settling into the cleft between FliG_N_ and FliG_C_. Structural models as in Movie 1.
