# Supplementary material for: Texas 2-step: a new model for YcgR::c-di-GMP action at the flagellar motor
Source: J Bacteriol. 2025 Nov 26;207(12):e00353-25. doi: 10.1128/jb.00353-25 (PMC12713396; doi:10.1128/jb.00353-25)
Supplement: Tables S1 and S2 — Strains, plasmids, and primers. [file jb.00353-25-s0002.docx]

**Table S1. Strains and Plasmids**

| **Strain** | **Genotype/Description** | **Source/reference** |
| --- | --- | --- |
| MG1655 | Wild type *E. coli* F^-^ λ^-^ *ilvG*- *rfb*-50 *rph*-1 | Lab collection |
| NBN28 | MG1655 ∆*fliC* | This work |
| NBN77 | NBN28 + ∆*pdeH* ∆*ycgR* | This work |
| NBN47 | NBN77 ∆*cheZ*+ pFD313 + pSEVA224 | This work |
| NBN48 | NBN77 + pFD313 + pSEVA224 | This work |
| NBN65 | NBN28 *fliG* (∆169-171) | This work |
| NBN68 | NBN77 ∆*cheY* + pFD313 + pSEVA224 | This work |
| NBN89 | NBN65 ∆*pdeH* ∆*ycgR* + pFD313 + pSEVA224 | This work |
| HCB5 | AW405 ∆*fliC* | Scharf Lab, (1) |
| NBN116 | HCB5 ∆*pdeH* + pFD313 + pSEVA224 | This work |
| NBN121 | NBN116 *fliG* (∆169-171) + pFD313 + pSEVA224 | This work |
| AB434 | MG1655 ∆*ycgR::Frt* | (2) |
| AB607 | MG1655 ∆*pdeH::Frt* | (2) |
| AB1468 | AB607 1976787::Tn*mariner*(kan) *motA*-1 (G93E) | (2) |
| AB1576 | AB607 1976787::Tn*mariner*(kan) *motA*-4 (G93R) | (2) |
| AB1577 | AB607 1976787::Tn*mariner*(kan) *motA*-2 (S96L) | (2) |
| AB1578 | AB607 1976787::Tn*mariner*(kan) *motA*-3 (G93V) | (2) |
| JP1501 | AB1577 ∆*ycgR* | This work |

| **Plasmid** | **Expressed Protein** | **Host Plasmid** | **Resistance** | **Induction** | **Reference** |
| --- | --- | --- | --- | --- | --- |
| pCP20 | FLP recombinase | **N.A*** | Ampicillin | Constitutive | (3) |
| pSEVA224 | YcgR | N.A. | Kanamycin | IPTG | (4) |
| pFD313 | FliC^sticky^ | pTRC99a | Ampicillin | IPTG | (5) |
| pMA7CR_2.0 | λ Red β-protein and Cas9 | pMA7 | Ampicillin | L-arabinose and **aTc*** | (6) |
| pMAZ-SK | gRNA | pCOLA-duet | Kanamycin | aTc | (6) |
| pBAD24 | Cloning vector | N.A | Ampicillin | L-arabinose | (7) |
| pVN8 | YcgR-GFP | pBAD24 | Ampicillin | L-arabinose | (8) |

**N.A***: Not applicable

**aTc***: Anhydrotetracycline

**Table S2. Primers pairs for gRNA and repair oligonucleotide for FliG deletion**

| **Name** | **Sequence** |
| --- | --- |
| 1_Fwd | TAGTGGCTGGCTGCACGCCGCCAA |
| 1_Rev | AAACTTGGCGGCGTGCAGCCAGCC |
| 2_Fwd | TAGTGTCAGCTCCGCCAGCGCGGC |
| 2_Rev | AAACGCCGCGCTGGCGGAGCTGAC |
| 3_Fwd | TAGTGAGCAAGCCATTCAGTACTT |
| 3_Rev | AAACAAGTACTGAATGGCTTGCTC |
|  |  |
| *fliG*_Repair oligos | CTGCGCCACGACGTGATGTTGCGTATCGCCACATTTGGCGGCGTGCAGCTGGCGGAGCTGACAGAAGTACTGAATGGCTTGCTCGACGGTCAGAATC |

**References**

1. Armstrong JB, Adler J, Dahl MM. 1967. Nonchemotactic mutants of Escherichia coli. J Bacteriol 93:390-8.

2. Boehm A, Kaiser M, Li H, Spangler C, Kasper CA, Ackermann M, Kaever V, Sourjik V, Roth V, Jenal U. 2010. Second messenger-mediated adjustment of bacterial swimming velocity. Cell 141:107-16.

3. Datsenko KA, Wanner BL. 2000. One-step inactivation of chromosomal genes in *Escherichia coli* K-12 using PCR products. Proc Natl Acad Sci U S A 97:6640-5.

4. Nieto V, Partridge JD, Severin GB, Lai RZ, Waters CM, Parkinson JS, Harshey RM. 2019. Under Elevated c-di-GMP in Escherichia coli, YcgR Alters Flagellar Motor Bias and Speed Sequentially, with Additional Negative Control of the Flagellar Regulon via the Adaptor Protein RssB. J Bacteriol 202.

5. Kuwajima G. 1988. Construction of a minimum-size functional flagellin of Escherichia coli. J Bacteriol 170:3305-9.

6. Ronda C, Pedersen LE, Sommer MO, Nielsen AT. 2016. CRMAGE: CRISPR Optimized MAGE Recombineering. Sci Rep 6:19452.

7. Guzman LM, Belin D, Carson MJ, Beckwith J. 1995. Tight regulation, modulation, and high-level expression by vectors containing the arabinose PBAD promoter. J Bacteriol 177:4121-30.

8. Paul K, Nieto V, Carlquist WC, Blair DF, Harshey RM. 2010. The c-di-GMP binding protein YcgR controls flagellar motor direction and speed to affect chemotaxis by a "backstop brake" mechanism. Molecular cell 38:128-39.
